# A *Theileria annulata* parasite with a single mutation, methionine 128 to isoleucine (M128I), in cytochrome B is resistant to buparvaquone

**DOI:** 10.1371/journal.pone.0299002

**Published:** 2024-04-16

**Authors:** Shahin Tajeri, Debasish Chattopadhyay, Gordon Langsley, Ard M. Nijhof

**Affiliations:** 1 Institute for Parasitology and Tropical Veterinary Medicine, Freie Universität Berlin, Berlin, Germany; 2 Veterinary Centre for Resistance Research, Freie Universität Berlin, Berlin, Germany; 3 Department of Medicine, University of Alabama at Birmingham, Birmingham, Alabama, United States of America; 4 Inserm U1016-CNRS UMR8104, Institut Cochin, Paris, France; 5 Laboratoire de Biologie Comparative des Apicomplexes, Faculté de Médecine, Université Paris Descartes—Sorbonne Paris Cité, Paris, France; Guru Angad Dev Veterinary and Animal Sciences University (GADVASU), INDIA

## Abstract

Tropical theileriosis is a fatal leukemic-like disease of cattle caused by the tick-transmitted protozoan parasite *Theileria annulata*. The economics of cattle meat and milk production is severely affected by theileriosis in endemic areas. The hydroxynaphtoquinone buparvaquone (BPQ) is the only available drug currently used to treat clinical theileriosis, whilst BPQ resistance is emerging and spreading in endemic areas. Here, we chronically exposed *T*. *annulata*-transformed macrophages *in vitro* to BPQ and monitored the emergence of drug-resistant parasites. Surviving parasites revealed a significant increase in BPQ IC_50_ compared to the wild type parasites. Drug resistant parasites from two independent cloned lines had an identical single mutation, M128I, in the gene coding for *T*. *annulata* cytochrome B (*Tacytb*). This *in vitro* generated mutation has not been reported in resistant field isolates previously, but is reminiscent of the methionine to isoleucine mutation in atovaquone-resistant *Plasmodium* and *Babesia*. The M128I mutation did not appear to exert any deleterious effect on parasite fitness (proliferation and differentiation to merozoites). To gain insight into whether drug-resistance could have resulted from altered drug binding to TaCytB we generated *in silico* a 3D-model of wild type TaCytB and docked BPQ to the predicted 3D-structure. Potential binding sites cluster in four areas of the protein structure including the Q_01_ site. The bound drug in the Q_01_ site is expected to pack against an alpha helix, which included M128, suggesting that the change in amino acid in this position may alter drug-binding. The *in vitro* generated BPQ resistant *T*. *annulata* is a useful tool to determine the contribution of the various predicted docking sites to BPQ resistance and will also allow testing novel drugs against theileriosis for their potential to overcome BPQ resistance.

## Introduction

Tropical theileriosis (*Theileria annulata* infection) is among the major tick-borne diseases of cattle in the tropics with significant impact on livestock production [1]. In endemic countries it is a problem of small holder farmers and traditional cattle production systems that rely on pasture grazing to feed their livestock, where exposure to ticks is a risk factor. Cancer resembling hyperproliferation and widespread tissue dissemination of monocytes, macrophages and B cells infected and transformed by *T*. *annulata* are the major cause of the disease that manifests with high fever, swelling of lymph nodes, cachexia and hemorrhage in mucosal membranes [2]. Further development and access of the parasites to the blood stream is associated with anemia and jaundice, whose pathogenesis remains largely unknown as intracellular replication of blood forms (i.e. piroplasms) is very limited [3]. Tropical theileriosis is usually a fatal disease if untreated and animals affected sub clinically have reduced productivity and act as reservoir for tick transmission.

Akin to many other vector-borne diseases, control of tropical theileriosis is not straightforward. However, availability of a live attenuated vaccine and a drug has successfully enhanced disease control in some areas [4]. Although the attenuated vaccine provides good immunological protection against field parasite challenge, the logistics of mass production and field deployment make it unpractical to produce for many countries. In such countries, theileriosis control largely depends on administration of anti-Theilerial hydroxynaphtoquinone, commercially known as buparvaquone (BPQ), to affected animals.

The antimalarial drug atovaquone, a substituted hydroxynaphtoquinone, is known to target *Plasmodium* cytochrome B [5] and cause a depolarization in mitochondrial membrane potential [6] that impairs energy and nucleic acid production (pyrimidine biosynthesis) resulting in rapid parasite death [7]. BPQ, although considered as a naphtoquinone, structurally differs from atovaquone and its mechanism of action is not well understood. Resistance conferring mutations of cytochrome B that occur in response to atovaquone treatment have been observed in closely related parasites *P*. *falciparum* [8], *P*. *berghei* [9], *Babesia gibsoni* [10, 11] and *Toxoplasma gondii* [12]. Due to some reports of *T*. *annulata* cytochrome B nucleotide polymorphisms associated with BPQ treatment failure [13–15], it has been suggested that similar to atovaquone, BPQ also targets cytochrome B. In transforming *Theileria* species there is evidence that BPQ is able to additionally inhibit a secreted parasite enzyme (TaPin1) that targets intracellular signal transduction pathways associated with cellular metabolism [16] and oncogenesis [17]. In addition, there is increasing evidence of BPQ being effective against parasites other than *Theileria*. Examples include inhibition of *Besnoitia besnoiti* tachyzoite proliferation [18], blockade of vertical transmission of *Neospora caninum* in a mouse model of neosporosis [19], anti-Leishmanial activity [20, 21], killing the hydatid worm *Echinococcus granulosus* metacestodes [22] and even fungi e.g. Sporothrix sp. [23]. This range of activities towards a wide spectrum of pathogens suggests that the drug targets a conserved enzyme common to the many species.

To combat BPQ resistance, there is a growing search for possible BPQ replacement therapies and several compounds have shown to be active against *Theileria* parasites [24–26]. However, these molecules are far from entering the market leaving BPQ as the only choice for field veterinarians to treat clinical theileriosis. Unfortunately, one study has shown tick transmissibility of BPQ-resistant *T*. *annulata* parasites [27] and another detailed investigation found increasing prevalence of cytochrome B mutations associated with BPQ resistance in the field [28]. The Hacilarilioglu et al study reported that BPQ selection pressure results in reduced clonal diversity of parasite genotypes within the host. However, except for one report [24] there is no direct evidence that cytochrome B mutations that arise in response to BPQ treatment actually confer drug-resistance. Therefore, the aim of the current work was to examine if upon lethal exposure to BPQ mutations occur in the cytochrome B gene of cloned *T*. *annulata*. We show that transformed macrophages harboring BPQ resistant parasites proliferate at the same rate as transformed macrophages harboring wild type parasites. Furthermore, schizonts of cytochrome B mutant parasites could differentiate to merozoites when culture temperatures are increased. The *in vitro* generated BPQ-resistant *T*. *annulata-*infected line constitutes a valuable tool to study several aspects of drug-resistance including vectorial transmissibility and development of novel therapies active against BPQ resistance in the field.

## Results

### *Theileria annulata* cytochrome B gene mutates under buparvaquone selection

**Fig 1:**
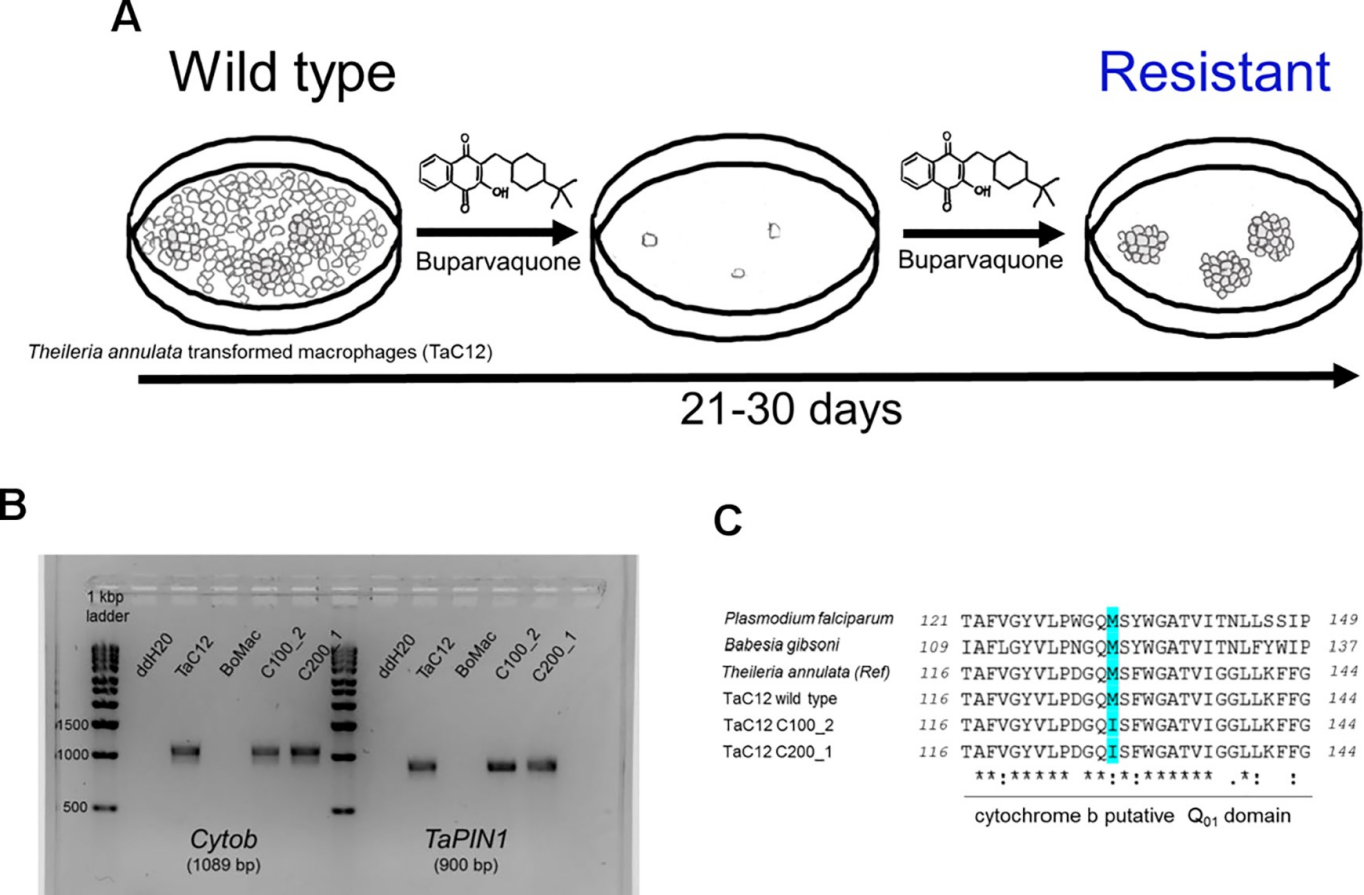
Selection and genetic characterization of buparvaquone-resistant *Theileria annulata* (*Ta*M128I). **(A)** Schematic figure showing the procedure for *in vitro* generation of BPQ resistance *Theileria annulata*. Briefly, adherent TaC12 cells were exposed to optimal BPQ dose (100 nM) and twice the optimal dose (200 nM) in two independent culture flasks. BPQ was refreshed every 72 h until resistant clones appeared in 3–4 weeks. Clones were then picked and expanded for further characterization. **(B)** Agarose gel electrophoresis image of *Tacytb* (1089 bp) and *Tapin1* (900 bp) PCR products. BoMac is a virus transformed bovine macrophage cell line used as negative control for PCR. **(C)** Protein sequence alignment of putative Q_01_ BPQ binding domain of *T*. *annulata* cytochrome B (116–144 aa) from *Plasmodium falciparum* (PF3D7_MIT02300), *Babesia gibsoni* (WAS37240.1), *T*. *annulata* (Reference PiroplasmaDB ID: Tap370b08.q2ca38.03c), wild type TaC12 and two independent BPQ-selected clones (TaC12 C100_2 and TaC12 C200_1). BPQ selection pressure resulted in a single mutation (G to A) at nucleotide 387 of *cytb* resulting in methionine 128 to isoleucine (M128I) amino acid substitution. No mutations were detected in *Tapin1*. M128 in *T*. *annulata* corresponds to M133 in *Plasmodium* and M121 in *B*. *gibsoni*. M133I and M121I mutations of cytochrome B confer resistance to atovaquone (a BPQ related chemical) in *Plasmodium* and *Babesia*, respectively.

To generate BPQ resistant *T*. *annulata*, a cloned *T*. *annulata* transformed macrophage line (TaC12) was exposed to 100 nM and 200 nM doses in independent cell culture flasks. These doses were chosen based on reported effective *in vitro* doses of 91.9 nM [29] and 153 nM [30]. BPQ was refreshed every 72 h and cultures were monitored for possible emergence of resistant infected macrophages. Confirming that the clones of TaC12 line used in this study behaved as wild type, BPQ treatment eliminated over 99% of transformed macrophages during the first 72–144 h. However, continuing culture in the presence of 100 nM and 200 nM of BPQ led to emergence of small number of colonies (**Fig 1A**) 3–4 weeks post initial drug administration. One individual clone from each dose group was picked and expanded. Since BPQ resistance has been associated with nucleotide mutations (SNPs) in parasite cytochrome B (*Tacytb*, Gene ID: Tap370b08.q2ca38.03c) [13, 14, 28, 31] and *T*. *annulata* prolyl isomerase 1 (*Tapin1*, Gene ID: TA18945) [17, 32], both *Tacytb* and *Tapin1* were PCR amplified from the wild-type BPQ-susceptible TaC12, and from the clones that emerged under 100 nM and 200 nM BPQ pressure (**Fig 1B**). PCR products were Sanger sequenced and compared to the reference *T*. *annulata* Ankara *cytb* and *pin1* gene sequences. Interestingly, a single G-to-A mutation at position 387 of *Tacytb* could be detected in both drug-resistant clones. No mutations in *Tapin1* were detected in BPQ-selected parasites. *Tacytb* and *Tapin1* sequences of wild type TaC12 were 100% identical to the reference Ankara gene sequences. The mutation found in *Tacytb* following BPQ exposure was located within the predicted ubiquinone biding site of cytochrome B (Q_01_) that includes residues 116 to 144 of TaCytB (PiroplasmaDB ID: Tap370b08.q2ca38.03c) [28]. The ATG to ATT change led to a non-synonymous amino acid codon substitution of methionine to isoleucine at codon 128 (M128I). M128 in TaCytB corresponds to *P*. *falciparum* M133 and *Babesia gibsoni* M121 (**Fig 1C**). Thus, like atovaquone-resistant *P*. *falciparum* BPQ-resistant *T*. *annulata* displays similar mutations in parasite CytB as atovaquone-resistant *P*. *falciparum* [8].

### Experimental validation of *in vitro* generated buparvaquone resistant *Theileria annulata*

**Fig 2:**
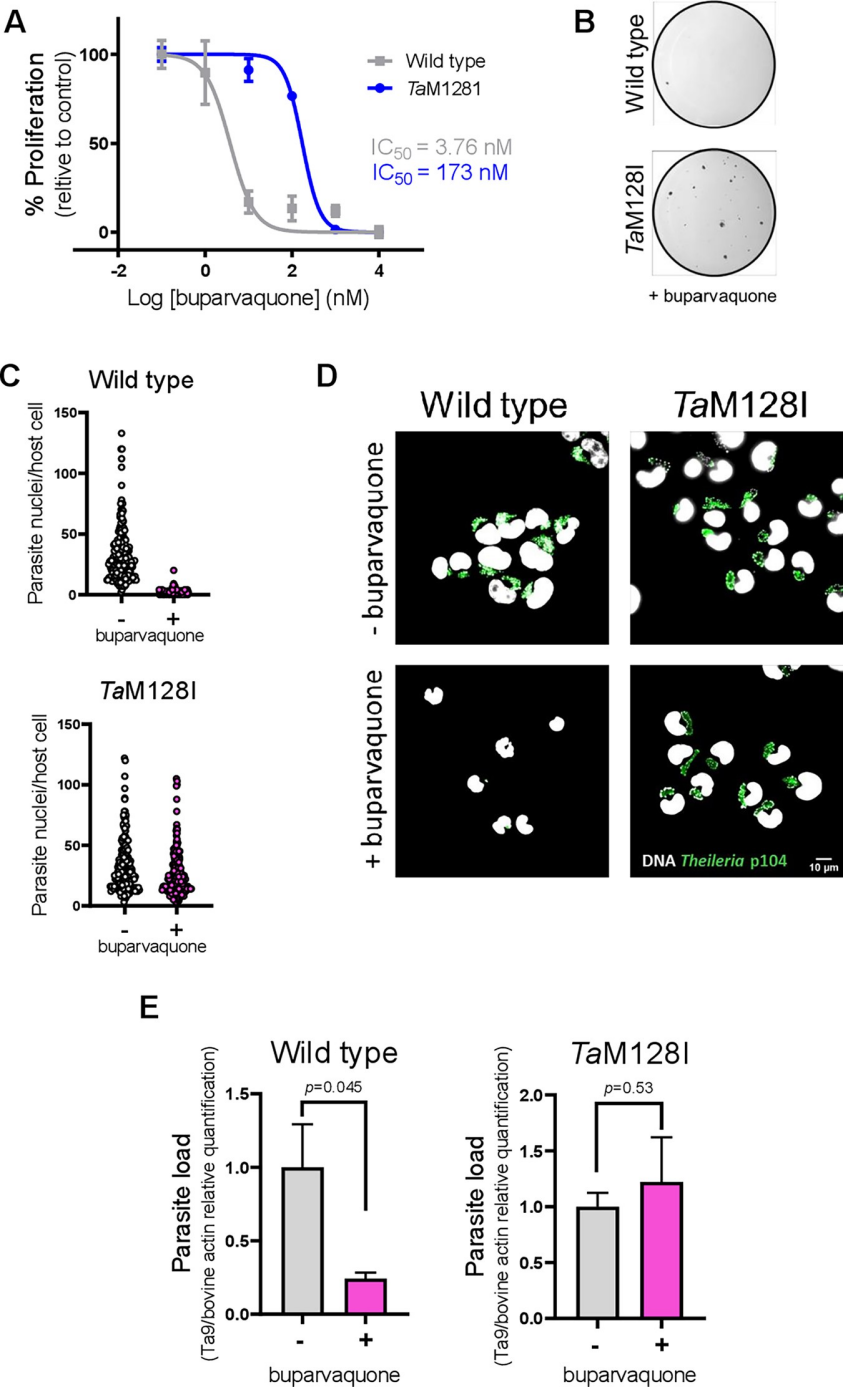
*Theileria annulata* parasites with M128I mutation in cytochrome B (*Ta*M128I) resist buparvaquone treatment. **(A)** Proliferation dose-response curves of wild type and *Ta*M128I containing macrophages treated for 72 h with 1, 10, 100, 1000 and 10000 nM BPQ. A clear right shift in dose-response curve resulted from decreased susceptibility of macrophages transformed by *Ta*M128I parasite compared to the wild type is detectable. **(B)** *Ta*M128I containing TaC12 cells form colonies in soft agar following BPQ treatment whereas the wild-type TaC12 did not. **(C)** Wild type and *Ta*M18I parasite containing cells were grown on coverslips and treated with BPQ for 72 h. The cells were then fixed and stained with DAPI to visualize and manually quantify the number of parasite nuclei per infected cell. The mean number of parasite nuclei per infected cell sharply decreased to zero nuclei (due to parasite death and elimination) when wild type TaC12 was treated with BPQ, but no obvious drop in nuclei number was seen in *Ta*M128I containing cells. Results represent pooled data from two independent experiments each quantified nuclei in 100 infected cells. **(D)** Immunofluorescence images of TaC12 macrophages containing wild type or cytochrome mutant *Ta*M128I parasites. The white signal shows host and parasite DNA and the green signal shows *Theileria* schizont membrane (detected by p104 cell surface marker). Up to 50% of wild type cells completely lost *T*. *annulata* schizonts when exposed for 72 h to BPQ, but no p104 reduction happened in *Ta*M128I parasites. **(E)** qPCR quantification of single copy *Theileria* gene *Ta9* (TA15705) in genomic DNA isolated from wild type macrophages and macrophages containing mutant *Ta*M128I parasites in absence or presence of drug. Relative copy numbers of *Ta9* significantly reduced upon BPQ mediate parasite killing in wild type TaC12, but was not affected in cells with *Ta*M128I parasites. All experiments where repeated twice (*n = 2*). The results represent mean ± standard deviations. Significance was estimated by student’s t test. BPQ treatment dosage was 100 nM for 72 h in B-E.

Since BPQ selection resulted in parasites with a mutation in cytochrome B, we examined if *T*. *annulata* parasites with the M128I mutation in cytochrome B (named herein *Ta*M128I) do indeed display reduced susceptibility to BPQ treatment. To test this, we compared the proliferation of cloned parasites that survived 100 nM BPQ treatment (TaC12 C100_2) to that of parental wild type infected macrophages. Macrophages harboring *Ta*M128 parasites displayed a right shift in the dose-response curve and more than 40-fold higher IC_50_ value (**Fig 2A**). Next, following 72 h treatment with 100 nM both resistant and wild type infected macrophages were grown in soft agar for 21 days.

*Ta*M128I-infected macrophages formed colonies in soft agar, while wild type parasite infected macrophages did not (**Fig 2B**). Each *T*. *annulata*-transformed macrophage contains a single parasite schizont as a syncytium of several nuclei. The number of nuclei per syncytium is an indicator of parasite fitness [17, 24]. BPQ treatment caused a drastic drop in the number of nuclei per syncytium in wild type infected macrophages, whereas no obvious reduction in the number of parasite nuclei per syncytium was observed for *Ta*M128I infected macrophages (**Fig 2C**). In addition, BPQ treatment eliminated parasites in the wild type group (as detected by immunofluorescence staining for schizont membrane marker protein p104), but all *Ta*M128I-infected macrophages stained with the p104 monoclonal antibody (**Fig 2D**). Similarly, the copy number of the unique parasite gene *Ta9* (TA15705) significantly diminished in wild type infected, but not in *Ta*M128I-infected macrophages (**Fig 2E**). Taken together, both proliferation and parasite quantification confirmed that *Ta*M128I-infected macrophages are resistant to BPQ.

### Does the M128I mutation and *in silico* predicted docking site of buparvaquone overlap?

**Fig 3:**
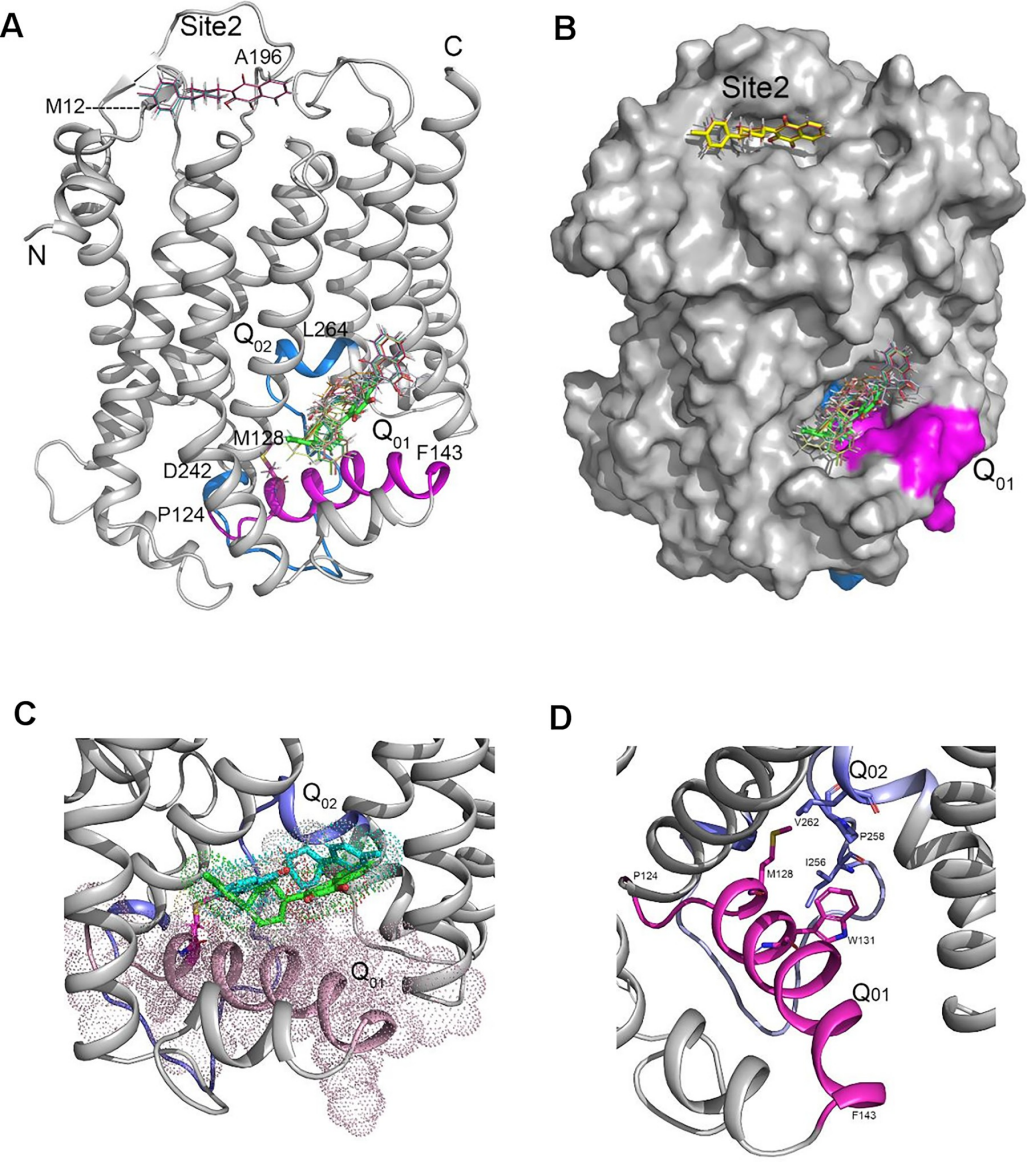
3-D Structural model for TaCytB. **(A)** 3-D structure for TaCytB was predicted using the RoseTTaFold on Robetta server. A cartoon drawing of the model is shown in grey. The N and C termini are labeled. M128 residue is shown as stick model and labeled. The structural model was used for docking BPQ using SwissDock [33], which predicted 257 docking poses distributed in four sites on the protein structure (S1 Fig). Among the 25 top ranking poses, 22 are located in the Q_01_ site, which spans residues 124–143 (shown in light magenta) and includes the helix α9. The remaining 3 poses (out of top 25) are predicted to bind to a pocket near the N-terminus. This site is labeled Site2. The Q_02_ site is colored blue. **(B)** Surface drawing showing the binding pockets for top 25 poses. **(C)** BPQ within the binding pocket. Close up view of the two top ranking docking poses of BPQ in the TaCytB binding pocket. Poses (#1 in cyan and #9 in green) are shown as stick model. Site Q_01_ is shown in light magenta and the Q_02_ site is depicted in light blue. Dots representing van der Waals sphere are shown for the residues in Q_01_ site and the drug molecules. **(D)** Environment of M128 residue. Cartoon diagram showing the Q_01_ (light magenta) and Q_02_ sites (light blue) labeled. The side chain of M128 sticks into a hydrophobic environment provided by the aliphatic and aromatic side chains of residues W131, I256, P258 and V262.

One way that M128I-parasites may have become resistant to BPQ is that the change in amino acid negatively impacts on drug binding to TaCytB. To test this notion we generated 3D-model of TaCytB (**Fig 3A***)*. BPQ was docked onto the predicted model structure using SwissDock program [33], which predicted 257 different docking poses for BPQ clustered at four different areas on the TaCytB model (**S1 Fig**). The poses differ in the location of the binding sites, and the conformation and orientation of BPQ. These poses, even when bound in the same area or pocket, display different interactions between the drug and the target. While many of the poses likely represent surface interactions, some binding poses are located in distinct pockets within the protein structure. When all poses were sorted based on overall binding efficiency (deltaG, the predicted binding site for the 22 of the top 25 poses are found to be located in the same area previously described as the atovaquone binding site Q_01_ in *P*. *falciparum* CytB [8] (**Fig 3A** and **3B**; see **S1 Table** for deltaG values). The remaining 3 poses (of the top 25) bind in a pocket near the N-terminus of the TaCytB molecule (Site 2 in **Fig 3A** and **3B**). The docked BPQ molecules bound in the Q_01_ site are expected to form van der Waals and hydrophobic interactions with TaCytB residues including those within the α9 helix (residues 127–143 in TaCytB) (**Fig 3C**) [8]. In the TaCytB structural model, residue Met128 is located near the N-terminal end of the α9-helix with its side chain protruding in a hydrophobic environment contributed by Trp131 (Q_01_) and Ile256, Ile257, Pro258 and Leu262 side chains (Q_02_) (**Fig 3D**). Therefore, M128I mutation may alter the local structure that affects BPQ binding to the Q_01_ site.

### Buparvaquone-resistant *Theileria annulata*-transformed macrophages are still able to produce merozoites

**Fig 4:**
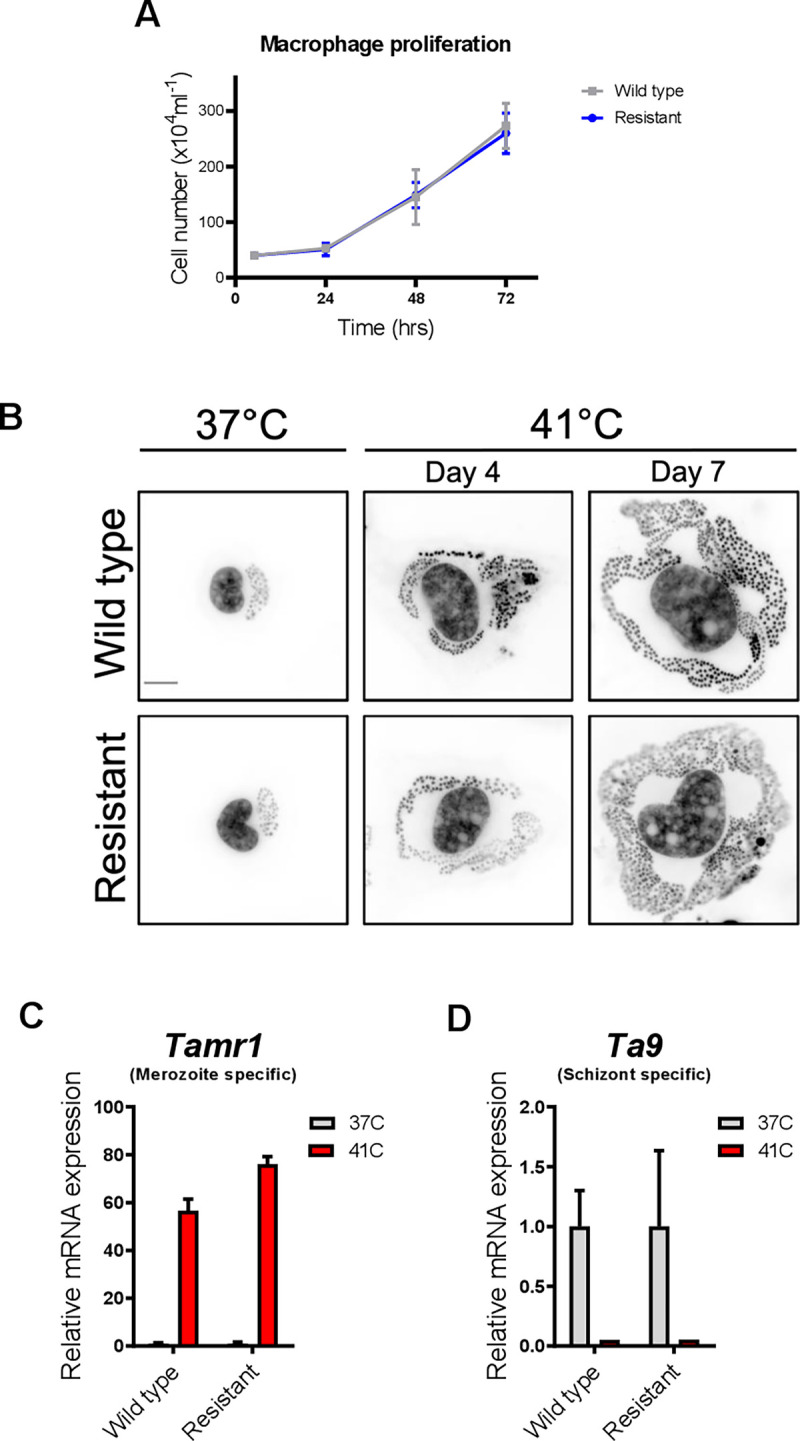
Cell proliferation and parasite differentiation in macrophages containing wild type or buparvaquone resistant *Ta*M128I parasites. **(A)** Proliferation curves of bovine macrophages having either wild type or BPQ resistant parasites showing same multiplication rates. **(B)** To induce merogony, *Theileria* transformed macrophages were cultured at 41°C for 7 days on cover slips. The cells were fixed at day-4 and day-7 post seeding, stained with DAPI dye and subjected to fluorescence imaging. The black and white inverted images depict that both wild type and BPQ resistant parasites gradually increase their nuclei when grown at 41°C. **(C)** and **(D)**, qRT-PCR expression measurement of a merozoites specific gene *Tamr1* and a schizont specific gene *Ta9* following 7 days culture at 41°C. Seven days culturing at 41°C caused an upregulation of *Tamr1* and downregulation of *Ta9* in wild type and BPQ resistant parasites. Results are representative of independent experiments (*n = 2*). mRNA levels were normalized to *T*. *annulata* actin II (= TA13410). Significance in mean differences of control and treatment groups was estimated by two-tailed student’s t test. Mean fold change in mRNA expression level ± standard deviations shown.

We have shown above (**Fig 4A**) that both M128I- and wild type-infected macrophages proliferate at the same rate in the absence of drug pressure indicating that there is no fitness cost for harboring the M128I mutation. However, atovaquone-resistant *Plasmodia* (*P*. *falciparum* and *P*. *berghei*) harboring *cytb* mutations fail to develop from oocysts to sporozoites in mosquitoes, and consequently drug-resistant parasites are not spread by mosquitoes [34]. For this reason, we tested if the M128I mutation negatively impacts on schizont to merozoites differentiation, since merozoite-infected red blood cells are taken up and are infectious to ticks. To this end, wild type and BPQ-resistant TaC12 macrophages were cultured at 41°C for 7 days to induce merozoite production [35]. The significant increase in parasite nuclei per infected cell, reduced expression of schizont specific gene *Ta9* and induction of merozoite marker *Tamr1* [35] following the temperature upshift showed that BPQ resistant *Ta*M128I similar to wild type schizonts are able to produce merozoites (**Fig 4B** and **4C**). Therefore, the M128I mutation in TaCytB B does not appear to hinder merozoite production suggesting that ticks feeding on cattle infected with *Ta*M128I might spread BPQ resistance in the field.

## Discussion

Since its first discovery and use in 1985 against theileriosis [36], BPQ has been highly effective in controlling *T*. *annulata* and *T*. *parva* infections. The first clinical report of BPQ-resistant *T*. *annulata* was in 2010 from Tunisia [27], and increasing cases of resistance throughout the world have been reported since [13–15]. A recent Turkish study showed that the rate of resistance associated mutations increased in the field from 1998 to 2012 [28] and thus, it is feared that BPQ resistance may become (or might already be) a major issue in tropical theileriosis control programs in the future.

BPQ resistance in the field is associated with clonal selection of specific parasite genotypes [28]. We therefore used a cloned *T*. *annulata*-transformed macrophage cell line (TaC12) harboring a single parasite genotype to test if parasites can mutate their *cytb* gene under BPQ selection pressure. We observed that a minor population of infected macrophages survived drug-treatment and clones that appeared four weeks later had a single methionine 128 to isoleucine (M128I) mutation in TaCytb, but no mutation in TaPin1. M128>II28 mutation has not yet been reported for BPQ-resistant *T*. *annulata* field strains. However, amino acid mutations in TaCytB adjacent to M128 such as S129G and V135A have been frequently reported in BPQ-resistant field strains [13–15, 28]. It has been proposed that reactive oxygen species (ROS) production in mitochondria increases susceptibility of mitochondrial genes to spontaneous mutation [37]. Indeed, *Tacytb* is so polymorphic that it is often used to type field isolates, consistent with infection-induced ROS production [38, 39] contributing to emergence of *T*a*cytb* mutations. Recently, another BPQ-resistant *Tacytb* mutation was generated *in vitro* via incrementally increasing drug treatment of a *T*. *annulata-*infected bovine B cell line (TBL3) [24]. Similar to the M128I mutation described herein, under drug pressure two additional mutations M227V and A254T also emerged [24]. The simultaneous emergence of two additional *Tacyt*b mutations might be explained by the higher dose of BPQ (200 ng/ml = 612 nM) used to generate BPQ-resistant TBL3 [24]. However, since *Tacytb* readily acquires SNPs the contribution of M227V and A254T to BPQ resistance remains to be demonstrated and this is true for many of the *Tacytb* mutations reported for drug-resistant field isolates. **S2A Fig** shows the TaCytB model with the Q_01_ and Q_02_ sites colored in light magenta and light blue respectively. The boundaries of the sites are assigned based on the pairwise alignment of TaCytB and PfCytB sequences and following the assignment by [8]. We have mapped the locations of all reported BPQ mutation sites [13–15, 28, 31] on our model as shown in **S2B Fig**. As shown in **S2B Fig**, the majority of mutations are observed within or near the Q_01_ and Q_02_ sites, but at least one mutation (V227M) outside these sites has also been reported. Thus far no mutation has been reported in Site 2 (see Fig 3A and 3B) identified in our docking exercise although the docking poses were among the high-ranking group (ranks 4, 9 and 21). However, none of the docking sites corresponds to the location of V227.

Interestingly, geographically distinct strains of *Plasmodium* display different emergence frequencies of resistance to atovaquone [40] and distinct *cytb* mutations [41]. When TaCytB and *P*. *falciparum* (PfCytB) are aligned M128 in TaCytB corresponds to M133 in *P*. *berghei* and *P*. *falciparum*. Moreover, chronic exposure of *P*. *berghei*-infected mice or *P*. *falciparum*-infected red blood cell cultures to atovaquone frequently resulted mutation of methionine 133 to isoleucine (M133I) [8, 9, 34, 42]. Furthermore, atovaquone-resistant malaria parasites harboring this and other CytB mutations (*Pb*Y268N/C, *Pf*Y268S and *Pf*V259L) were unable to grow beyond the oocyst stage ablating parasite transmission by mosquitoes [34, 43]. However, a counter arguing study demonstrated menoctone-resistant *P*. *berghei* parasites with the M133I mutation could be transmitted by *Anopheles stephensi* mosquitoes [44]. Unlike the findings that atovaquone-resistant *Plasmodium* is non-transmissible by mosquito vectors [34, 43], BPQ-resistant *T*. *annulata* can be transmitted by ticks [27]. Furthermore, *Ixodes ricinus* ticks successfully transmitted ELQ-502-(a cytochrome B Q_02_ site targeting compound)-resistant *Babesia microti* with a mutation in *Bm*CytB (A218V) to mice [45]. In this context, we found that BPQ-resistant *T*. *annulata* parasites are able to produce merozoites, but it remains to be seen if *Ta*M128I parasites are able to complete their cycle in ticks and are infectious to cattle.

The existing reports of BPQ resistance transmission (in case of *T*. *annulata*) [27] and ELQ-502 resistant *Babesia* with mutated cytochrome B [45] highlights the idea that unlike *Plasmodium*, both *Babesia* and *Theileria* mitochondria might not be exclusively female inherited. Alternatively, it might suggest that drug-resistant and cytochrome-mutant *Theileria* and *Babesia* are less reliant on their mitochondrial respiration during tick stages (in contrast to *Plasmodium* in mosquitoes) and are thus able to complete their cycle in their arthropod vectors. One could speculate that apicomplexan parasites might have heteroplasmic mitochondrial genotypes, i.e. they are not all genetically identical. If true, a percentage of parasites harboring resistance-conferring cytochrome mutations (M128I in *Theileria* and M133I in *Plasmodium*) would fail to transmit, whereas a percentage of parasites harboring wild type mitochondria genomes would be transmitted. Support for apicomplexan heteroplasmy comes from the observation that ELQ-502-resistant mutant *Babesia* parasites developing in ticks were genetically unstable with a high rate of the wild-type allele emerging during the nymphal stage [45]. Examining the mutational status of *Tacytb* in *Ta*M128I parasites post-tick transmission would address the question of whether *Theileria* mitochondria are heteroplasmic.

## Conclusions

We were able to generate *in vitro* BPQ-resistant *T*. *annulata* from a cloned parasite population. BPQ-resistant parasites had a single M128I mutation in their cytochrome B that resembles atovaquone-resistant *Plasmodium*, *Babesia* and *Toxoplasma*. The M128I mutation did not appear to adversely affect parasite fitness (transformed macrophage proliferation and parasite differentiation to merozoites). Future studies will address tick transmissibility of M128I parasites and whether they can be used to evaluate novel drugs for their ability to treat BPQ-resistant tropical theileriosis.

## Materials and methods

### Cell culture and induction of merogony in *Theileria annulata* transformed macrophages

TaC12 is a cloned bovine macrophage cell line transformed by *T*. *annulata*. TaC12 was cultured in L-glutamine containing RPMI1640 (Gibco™) supplemented with 10% fetal bovine serum (Gibco™), 10 mM HEPES (Carl Roth), 100 U/ml penicillin/streptomycin (Gibco™) and passaged every 48 h (with the seeding density of 2–3 X 10^5^ cell/ml). BoMac is an SV40 virus transformed macrophage cell line. BoMac was cultured in DMEM/F-12 medium (Gibco™) supplemented 10% FBS and 100 U/ml penicillin/streptomycin. BoMac was passaged twice per week. Routine cell culture involved 25 cm^2^ vented cell culture flasks (VWR® International) that were kept at 37°C in a 5% CO2-in-air atmosphere incubator (Thermo Fisher).

To induce differentiation of *T*. *annulata* schizonts towards merozoites, cultures were incubated at 41°C. The 41°C cultures were passaged once at 48 h post transfer to an incubator set at 41°C and then kept at 41°C for 7 days.

### Generation of buparvaquone-resistant *Theileria annulata*

*Theileria annulata* transformed macrophages (TaC12) were treated for 3–4 weeks with 100 nM and 200 nM buparvaquone (MedChemExpress) doses in two independent T25 culture flasks. After one week of treatment almost all cells were killed by BPQ, but individual macrophage clones reappeared around four weeks after the start of the experiment. BPQ was refreshed every 72 h. When clones became visible they were picked up by a 200-μl pipette tip and transferred to individual wells of a 24-well plate (Sarsted AG & Co. KG) and kept until the wells become confluent and could further be passaged and tested. The ability of one clone (C100_2) to resist BPQ treatment was tested and compared to the wild type TaC12 in various tests.

### Cell proliferation assay and BPQ IC_50_ estimation

Cells from a confluent culture were counted and equally distributed in 12 or 24-well cell culture plates with or without BPQ. After 96 h post cell seeding, the cells were detached by trypsin (Gibco™) treatment and counted manually with an improved Neubauer® hemocytometer under an inverted light microscope (Motic AE2000). Cells numbers were reported as percentage of cells relative to the control non-treated group. To calculate IC_50_, a range of BPQ doses (1, 10, 100, 1000 and 10000 nM) were tested against bovine TaC12 macrophages and the percent inhibition of cell proliferation was calculated for each drug concentration normalized to the control. IC_50_ was then calculated in GraphPad prism version 5.

### Soft agar colony formation assay

Cells were initially cultured with or without BPQ for 72 h, washed once with PBS and counted. A two-layer low melting temperature agarose (Carl Roth, Germany) was prepared by a 1:1 dilution with 2X complete DMEM medium containing 20% FBS and 200 U/ml Penicillin/Streptomycin. First, the base acellular layer (1.5 ml of 1% percent agarose) was laid in each well of a six-well culture plate (Sarstedt, Germany). Following solidification of the base layer, the second cellular layer (1.5 ml of 0.7% agarose) including 10,000 cells per well was added. Finally, the second layer was covered with 1.5 ml complete RPMI1640 medium and was replenished twice per week. The cultures were grown for 21 days when visible colonies appeared. At this time point, cells were fixed and stained with 0.0001% crystal violet solution in methanol until all colonies (both superficial and deep colonies) were stained blue. Next, the wells were rigorously washed with distilled water until the background pale blue color of agarose gel turned colorless. This allowed imaging of the plates with an iPhone 12 on a white background. The images were transferred to Fiji image J software for further processing.

### DNA extraction, PCR and Sanger sequencing of *Tacyt* and *Tapin1* genes

DNA was prepared from10^6^ cells using NucleoSpin® DNA tissue XS kit (Macherey Nagel). PCR was done in a 50 μl reaction that included 1 unit S7 Fusion polymerase™ (Mobidiag Ltd.), 0,5 μM gene specific forward and reverse primers (Sigma Aldrich), 200 μM dNTPs and 1X high fidelity buffer. Amplification was done in an iQ™ PCR thermocycler (BIORAD) with the following program: Initial denaturation at 95°C for 1 min followed by 35 cycles of 10 sec denaturation at 98°C, annealing at 57°C for 15 sec, extension at 72°C for 45 sec. PCR was terminated by a final extension step at 72°C for 5 min. DNA from non-infected bovine macrophages (BoMac) and double distilled water served as negative control in PCR reactions. PCR products were resolved in 2% agarose (Biozym) gel at 90 V for 1 h and imaged by a Vilber Lourmat gel documentation system. The raw images were inverted in Fiji software and labelled in Microsoft Office Powerpoint. The rest of the PCR products were purified by a DNA Clean & Concentrator kit (Zymo research). Purified PCR products were dosed by Nanodrop1000 (ThermoFisher Scientific) and sent for Sanger sequencing to LGC genomics GmbH (Germany). DNA sequencing chromatograms were visualized in Chromas software. Cytb primers and pin1 primers were those described previously [17, 28].

### DAPI and *Theileria annulata* p104 (Ta-p104, Gene ID: TA08425) immunofluorescence staining

To count the number of schizont nuclei in each infected cell, cells were grown on round cover slips (10 mm diameter, Car Roth) in 24-well plates to sub-confluency in absence or presence of BPQ. Cells were then fixed by 4% paraformaldehyde (PFA) (Sigma) in PBS for 10–15 min. The cover slips were then fixed on microscope slide with a drop of mounting medium containing DAPI (ROTI®Mount FluorCare, Carl Roth, Germany). Parasite nuclei were quantified manually under a Leica DMi8 wide field microscope. To evaluate BPQ effect on *T*. *annulata* schizonts, BPQ treated or control TaC12 cells were first fixed with 4% paraformaldehyde (PFA) (Sigma-Aldrich) for 10 min and stained with mouse polyclonal anti-*Theileria* p104 antiserum (Gift of Brian Shiels, University of Glasgow, 1:1000 dilution in 0.4% Triton 100, 1% BSA in PBS) for 1 h. Following three PBS washes, Alexa Fluor 488 conjugated donkey anti-mouse secondary antibody (Invitrogen) (1:500 dilution in 0,1% Triton 100 in PBS for 1 h) was used to visualize p104 signal under a wide field fluorescent microscope (Leica DMi8). Presence of any residual p104 signal was considered as the cell being p104 positive. All staining steps were performed at room temperature. When necessary, images were taken by LASX PC software and finally processed in Fiji Image J.

### Quantification of parasite load by quantitative PCR (qPCR)

To quantify parasite load in BPQ treated and control cultures of wild type and BPQ resistant TaC12, DNA was extracted from cells with a NucleoSpin® DNA tissue XS kit (Macherey Nagel). The DNA was then quantified by Nanodrop and all sample concentrations were set to 10 ng/μl. The single copy *T*. *annulata* gene *Ta9* (= TA15705) was quantified from samples by qPCR in a 20 μl reaction. The PCR reaction included 0.5 μl of each forward and reverse primers at 10 μM, 10 μl SYBR green mix (Luna® Universal qPCR Master Mix, New England Biolabs), 2 μl DNA and 7 μl molecular grade water. PCR was done in C1000 Touch thermal cycler (BIORAD). *Ta9* levels were normalized to bovine 18S rRNA (NCBI accession ID: NR_036642.1). *Ta9* primers were Forward: CACAATGAATCTCCTAACATCTGG; Reverse: GCTCGTCTAATTAAACTCTTCT and bovine 18S rRNA primers were: Forward: TTCGATGGTAGTCGCTGTGC; Reverse: TTGGATGTGGTAGCCGTTTCT [46].

### Quantification of *Tamr1* (TA16685) and *Ta9* (TA15705) mRNA expression by quantitative real time qRT-PCR (qRT-PCR) in schizonts and merozoites

Total RNA was extracted from wild type and resistant parasite containing transformed macrophages cultured at 37°C (schizont) and cells cultured at 41°C for seven days (merozoites) by using NucleoSpin RNA plus (Macherey Nagel). 300 nanograms of RNA was converted to cDNA by Protoscript II first strand cDNA synthesis kit (New England Biolabs). cDNA from each sample was diluted 1:20 in DNase free water. The PCR reaction (20 μl) included 0.5 μl of each forward and reverse primers at 10 μM, 10 μl SYBR green mix (Luna® Universal qPCR Master Mix, New England Biolabs), 2 μl diluted cDNA and 7 μl molecular grade water. *Ta9* primers were forward: CACAATGAATCTCCTAACATCTGG; reverse: GCTCGTCTAATTAAACTCTTCT and Tamr1 primers forward: CCACTCCTGTAGCGGGTAAA; reverse: TTGTGGAGGTACTGACCCAAA). mRNA levels were normalized to *Theileria* actin II (TA13410) with the forward (GACATTAAGGAGCGGTGCTG) and reverse (AGTAGTGCCGTCTGGGAGTTT) primers [47]. The 2^−ΔΔCT^ Method was used to calculate relative mRNA expression levels.

### *In silico* docking of buparvaquone and *Theileria annulata* cytochrome B

To understand the effect of observed mutations on the potential of TaCytB to bind BPQ we generated homology models of TaCytB using RoseTTAFold (Robetta server: https://robetta.bakerlab.org/). This model is very similar to the one predicted by AlphaFold ((https://alphafold.ebi.ac.uk/). The root mean square deviation (*rmsd*) for superimposition of the two models is 1.1 Å; **S3 Fig**). We also used SWISS-MODEL program for prediction. The *rmsd* for superposition of our model and the one predicted by SWISS-MODEL is also 1.1 Å. The quality of the model was validated using the ERRAT program [48]. The Overall Quality Factor for our model is 97.18, and corresponding values for the AlphaFold and Swiss-Model generated models are 93.0 and 97.18, respectively. Good high-resolution structures generally produce values 95% or higher. Analysis of the stereochemical quality of the protein model using PROCHECK revealed that >99% of residues were within the favored regions of Ramachandran plot [49].

We then used our model for docking BPQ using the SwissDock [33] for prediction of potential binding sites for the drug on the TaCytB model using the default option without specifying any binding area on the target modelProtein structure and docking figures were made using PyMOL (PyMOL Molecular Graphics System, Version 1.3, Schrödinger, LLC.)

### Statistical analysis

Where necessary, student’s t test was used to assess significance. Estimated *p* values of less than 0.05 were considered statistically significant.

## Supporting information

S1 Fig Docking sites for BPQ on TaCytB structure. Cartoon drawing of TaCytB showing the areas of docked poses. N- and C-termini are labeled. The Q_01_ and Q02 sites are colored light magenta and blue and labeled. Majority of the top ranking poses bind within the Q_01_ site. A small number of high-ranking poses bind in the Site2 area.
(TIF)

S2 Fig Reported TaCytB mutations. **(A)** Cartoon drawing showing the Q_01_ and Q_02_ sites (in light magenta and light blue colors, respectively) in TaCytB structural model. N- and C-termini of the protein and each site are labeled. **(B)** Close up view of the location of reported BPQ mutation sites in TaCytB. While most sites are located within the Q_01_ and Q_02_ sites, one mutation outside these sites has been reported.
(TIFF)

S3 Fig AlphaFold comparison. Cartoon diagram showing superimposition of the TaCytB models generated by AlphaFold (AF-Q4UJ67-F1-model_v4 in cyan color) and by RoseTTa fold (grey). The rmsd for alignment of all Cα atoms is 1.1Å.
(TIF)

S1 Table Docking parameters. Binding parameters for different poses predicted for binding of buparvaquone in TaCytB structural model.
(PDF)

S1 Raw images (PDF)
