# Supplementary figures and images for: A Theileria annulata parasite with a single mutation, methionine 128 to isoleucine (M128I), in cytochrome B is resistant to buparvaquone
Source: PLoS One. 2024 Apr 16;19(4):e0299002. doi: 10.1371/journal.pone.0299002 (PMC11020719; doi:10.1371/journal.pone.0299002)

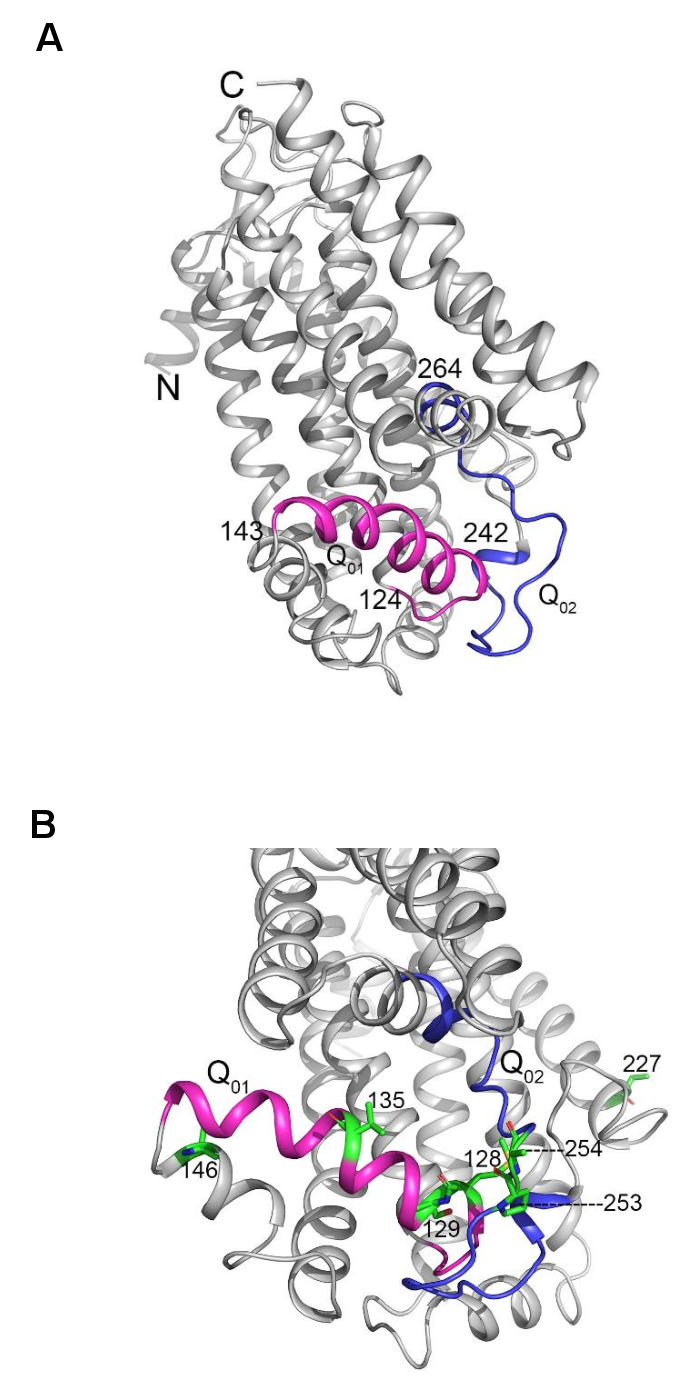

Supplement: S2 Fig — (A) Cartoon drawing showing the Q01 and Q02 sites (in light magenta and light blue colors, respectively) in TaCytB structural model. N- and C-termini of the protein and each site are labeled. (B) Close up view of the location of reported BPQ mutation sites in TaCytB. While most sites are located within the Q01 and Q02 sites, one mutation outside these sites has been reported. (TIFF) [file pone.0299002.s002.tiff]

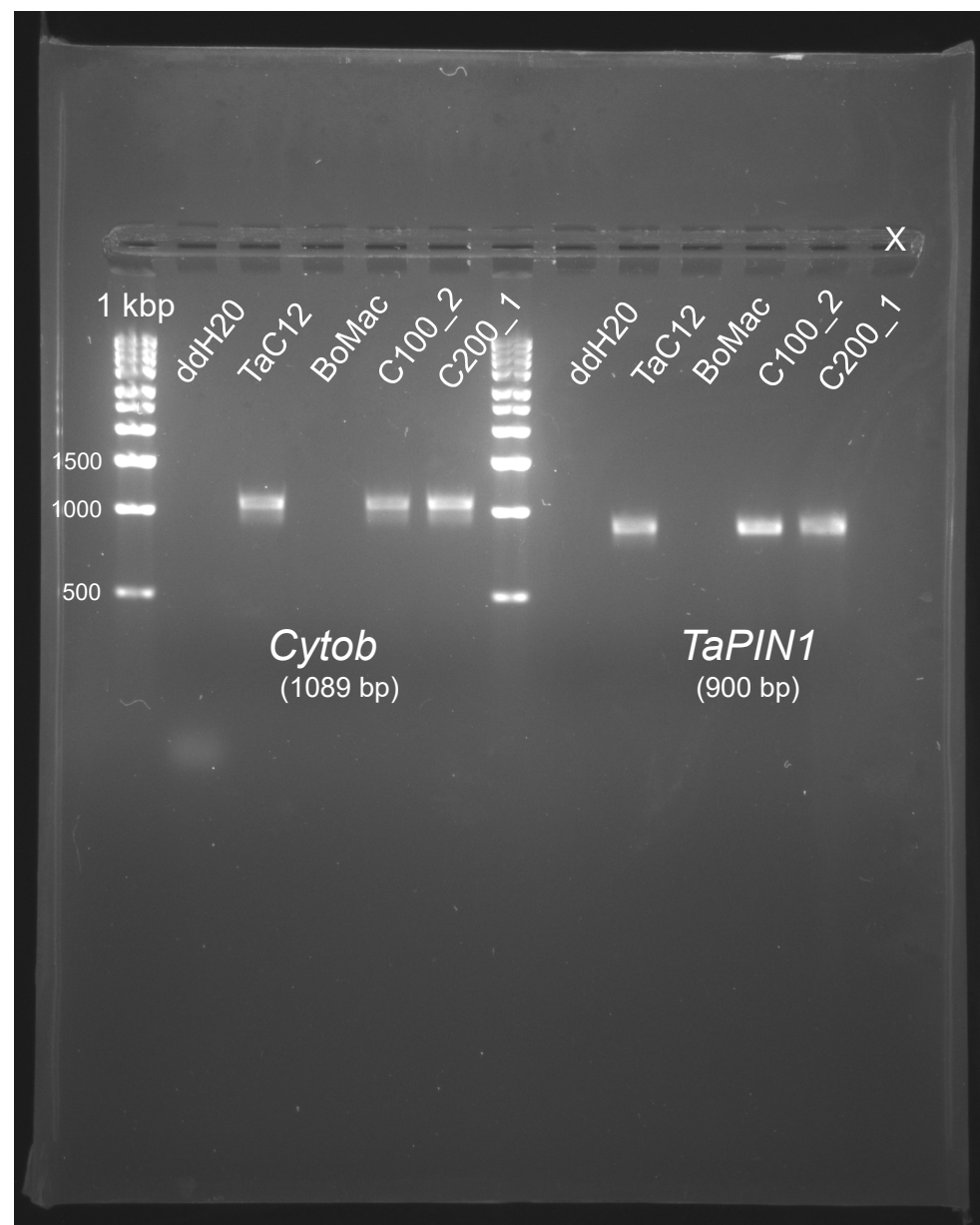

Supplement: S1 Raw images — (PDF) [file pone.0299002.s005.pdf]
