# Supplementary material for: A Theileria annulata parasite with a single mutation, methionine 128 to isoleucine (M128I), in cytochrome B is resistant to buparvaquone
Source: PLoS One. 2024 Apr 16;19(4):e0299002. doi: 10.1371/journal.pone.0299002 (PMC11020719; doi:10.1371/journal.pone.0299002)
Supplement: S1 Table — Binding parameters for different poses predicted for binding of buparvaquone in TaCytB structural model. (PDF) [file pone.0299002.s004.pdf]

Cluster#	Pose#	deltaG
12	0	-7.76475
12	2	-7.76014
12	1	-7.75127
10	0	-7.66098
1	1	-7.59538
1	0	-7.59307
1	2	-7.5627
10	1	-7.56205
24	0	-7.55489
8	3	-7.5472
8	4	-7.5472
8	5	-7.53717
8	0	-7.53538
8	1	-7.53282
8	2	-7.53282
1	5	-7.52882
4	0	-7.52527
4	1	-7.5225
24	1	-7.52124
24	2	-7.52124
10	2	-7.51556
8	6	-7.51342
3	1	-7.51206
3	0	-7.50767
1	6	-7.5038
4	3	-7.49654
4	4	-7.49599
3	3	-7.492
24	3	-7.45907
24	4	-7.45907
0	0	-7.45451
0	1	-7.45451
0	2	-7.45451
0	3	-7.45442
0	4	-7.45442
0	5	-7.45442
0	6	-7.45442
0	7	-7.45319
14	0	-7.44458
4	2	-7.42803

14	2	-7.4219
4	5	-7.42177
12	4	-7.41496
4	7	-7.40595
14	1	-7.39825
2	2	-7.3952
3	7	-7.39332
1	4	-7.3895
12	3	-7.38329
3	5	-7.3809
2	1	-7.37965
1	3	-7.37722
3	4	-7.37422
4	6	-7.36832
3	2	-7.36641
6	0	-7.35815
2	0	-7.35812
6	1	-7.3573
1	7	-7.34949
12	5	-7.33806
5	0	-7.32131
10	3	-7.30438
14	4	-7.28259
14	5	-7.27702
2	10	-7.26949
6	2	-7.26825
10	4	-7.26271
6	3	-7.24935
2	3	-7.24196
2	4	-7.24196
2	5	-7.24196
2	6	-7.24196
2	7	-7.24196
10	7	-7.24121
8	9	-7.24088
14	3	-7.2359
3	6	-7.22926
8	7	-7.22742
25	0	-7.22502
10	6	-7.22154
9	0	-7.22147

2	8	-7.21421
2	9	-7.21421
9	1	-7.21307
13	0	-7.20887
14	6	-7.20737
13	2	-7.20711
13	1	-7.20577
7	0	-7.204
10	5	-7.19356
7	1	-7.18583
23	4	-7.18091
23	5	-7.18091
23	6	-7.18091
23	7	-7.18091
11	0	-7.16065
11	1	-7.1539
8	8	-7.15218
7	2	-7.14664
15	0	-7.14643
15	1	-7.14643
22	0	-7.14627
22	1	-7.14627
15	6	-7.14382
29	1	-7.13899
15	3	-7.13807
15	4	-7.13355
15	5	-7.13355
15	7	-7.13098
15	2	-7.1292
17	0	-7.12833
23	10	-7.1273
7	5	-7.12082
22	2	-7.11644
22	3	-7.11644
22	4	-7.11644
17	1	-7.11204
7	3	-7.10286
23	9	-7.10154
6	4	-7.09567
17	4	-7.08918
16	3	-7.08687

7	4	-7.08392
16	1	-7.08345
29	2	-7.07283
17	7	-7.06413
29	0	-7.06335
17	5	-7.06177
23	2	-7.06098
23	3	-7.06098
29	3	-7.06086
6	5	-7.05166
16	0	-7.0448
21	12	-7.0397
6	9	-7.03352
6	7	-7.03321
16	2	-7.03234
17	3	-7.02794
13	3	-7.02758
6	6	-7.02584
2	11	-7.02525
16	7	-7.02305
17	6	-7.0155
2	12	-7.0054
17	9	-7.00355
2	13	-7.00119
20	1	-6.99505
20	0	-6.99367
16	4	-6.9935
16	5	-6.9935
13	4	-6.99269
29	4	-6.98734
26	9	-6.98034
17	8	-6.96267
16	6	-6.96181
20	2	-6.95739
6	8	-6.95571
17	2	-6.95151
29	5	-6.93256
7	6	-6.92742
14	7	-6.91254
7	7	-6.90996
29	7	-6.89839

22	5	-6.89456
22	6	-6.89456
22	7	-6.89456
25	1	-6.89321
2	14	-6.88262
23	0	-6.8819
23	1	-6.8819
0	8	-6.87806
21	10	-6.8729
26	0	-6.87005
26	1	-6.87005
26	2	-6.87005
19	1	-6.86456
21	13	-6.86359
21	14	-6.86009
18	0	-6.85759
18	1	-6.85759
18	2	-6.85759
18	3	-6.85759
21	2	-6.85414
21	3	-6.85414
21	4	-6.85414
21	0	-6.84186
21	1	-6.84186
21	9	-6.8415
19	0	-6.83943
18	5	-6.83269
18	6	-6.83269
18	7	-6.83269
29	6	-6.83269
21	11	-6.82948
26	3	-6.82896
21	5	-6.82774
21	6	-6.82774
21	7	-6.82774
26	4	-6.82538
26	5	-6.82538
26	6	-6.82538
26	7	-6.82538
0	9	-6.82205
6	10	-6.82004

19	3	-6.81239
18	4	-6.80513
21	8	-6.79947
19	2	-6.79739
31	1	-6.78813
31	2	-6.78813
31	3	-6.78515
31	4	-6.78515
13	5	-6.78415
6	13	-6.78389
23	8	-6.78075
31	0	-6.77742
6	11	-6.76653
28	6	-6.76176
6	12	-6.7569
28	4	-6.75021
28	3	-6.74844
11	2	-6.73386
32	6	-6.72938
31	6	-6.72539
31	5	-6.72342
28	2	-6.72246
28	5	-6.71695
19	5	-6.71292
19	4	-6.71257
28	1	-6.71104
28	0	-6.70211
32	4	-6.70068
32	1	-6.70062
32	0	-6.69418
32	2	-6.67992
32	3	-6.67032
32	5	-6.66664
13	7	-6.65449
26	8	-6.64319
30	1	-6.63324
30	0	-6.63138
13	6	-6.62263
19	6	-6.59886
28	7	-6.58991
27	0	-6.55226

27	1	-6.54701
31	7	-6.54674
32	7	-6.54569
27	2	-6.54326
27	6	-6.33778
27	4	-6.31997
27	3	-6.29949
27	5	-6.28255
27	7	-5.88768
33	0	118.4267
33	1	118.716
